# River ecosystem resilience to extreme flood events

**DOI:** 10.1002/ece3.4300

**Published:** 2018-07-24

**Authors:** Alexander M. Milner, Jessica L. Picken, Megan J. Klaar, Anne L. Robertson, Leonie R. Clitherow, Lawrence Eagle, Lee E. Brown

**Affiliations:** ^1^ School of Geography, Earth and Environmental Sciences University of Birmingham Birmingham UK; ^2^ Institute of Arctic Biology University of Alaska Fairbanks Alaska USA; ^3^ School of Biological and Chemical Sciences Queen Mary University of London London UK; ^4^ School of Geography & water@leeds University of Leeds Leeds UK; ^5^ Department of Life Sciences University of Roehampton London UK

**Keywords:** climate change, extreme floods, macroinvertebrates, meiofauna, recovery, resilience, salmonids

## Abstract

Floods have a major influence in structuring river ecosystems. Considering projected increases in high‐magnitude rainfall events with climate change, major flooding events are expected to increase in many regions of the world. However, there is uncertainty about the effect of different flooding regimes and the importance of flood timing in structuring riverine habitats and their associated biotic communities. In addition, our understanding of community response is hindered by a lack of long‐term datasets to evaluate river ecosystem resilience to flooding. Here we show that in a river ecosystem studied for 30 years, a major winter flood reset the invertebrate community to a community similar to one that existed 15 years earlier. The community had not recovered to the preflood state when recurrent summer flooding 9 years later reset the ecosystem back to an even earlier community. Total macroinvertebrate density was reduced in the winter flood by an order of magnitude more than the summer flood. Meiofaunal invertebrates were more resilient to the flooding than macroinvertebrates, possibly due to their smaller body size facilitating greater access to in‐stream refugia. Pacific pink salmon escapement was markedly affected by the winter flood when eggs were developing in redds, compared to summer flooding, which occurred before the majority of eggs were laid. Our findings inform a proposed conceptual model of three possible responses to flooding by the invertebrate community in terms of switching to different states and effects on resilience to future flooding events. In a changing climate, understanding these responses is important for river managers to mitigate the biological impacts of extreme flooding effects.

## 1 INTRODUCTION

Floods are a defining and natural feature of the flow regime of many rivers (Lake, 2000; Poff et al., 1997) but flood magnitude and frequency are increasing worldwide with climate change (Coumou & Rahmstorf, 2012; Milner, Robertson, McDermott, Klaar, & Brown, 2013) which will markedly alter their role in structuring riverine habitat and their associated biotic communities (Jones, 2013). Long‐standing debates remain unresolved regarding the relative importance of infrequent high magnitude floods versus the cumulative effects of more frequent lower magnitude events on fluvial geomorphology and associated biotic communities (Lewin & Macklin, 2010; Stanley, Powers, & Lottig, 2010). However, while there is a growing awareness that extreme climate events will modify riverine flows and associated habitats in which biological communities exist (Ledger & Milner, 2015), our overall understanding remains in its infancy (Coumou & Rahmstorf, 2012). Another key aspect of floods in addition to peak flow magnitude is their timing throughout the year, causing potentially different impacts, particularly with respect to biotic communities (George, Baldigo, Smith, & Robinson, 2015). Of particular significance is the need to understand the effects of contrasting flooding events on community resilience and assembly (George et al., 2015; Pearsons, Li, & Lamberti, 1992). We define resilience as incorporating two elements (a) resistance of the taxa to the initial disturbance and/or (b) ability of the taxa to recover rapidly (Holling, 1973). A key question is how communities reassemble following flooding events and whether this makes the community more resilient or less resilient to further change following a major event. In addition, a full understanding of the effects of extreme flooding events across a range of organismal groups has previously been hindered by the lack of long‐term predisturbance data to permit detailed insights into the interaction of community dynamics, successional processes, and river channel geomorphology (Poff et al., 1997).

**Figure 1:**
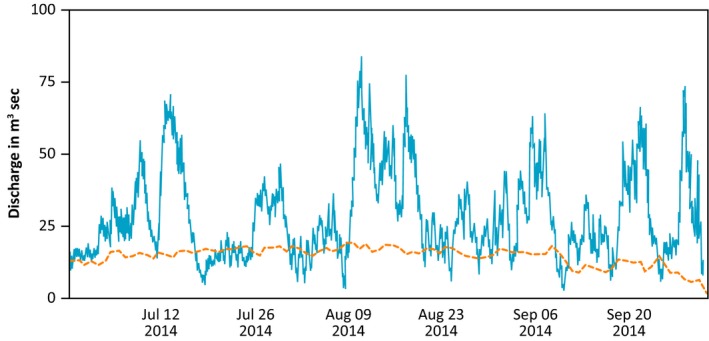
Discharge of Lemon Creek, Juneau, SE Alaska in 2014 with some events 8× median flow. Solid line = 2014 discharge; broken orange line = long‐term (30 yr) median

In southeast (SE) Alaska, the summer of 2014 saw record‐breaking prolonged high rainfall creating a series of large, recurrent, and atypical flood events during the summer/early autumn. At Bartlett Cove (SE Glacier Bay) June (133 mm) and July (211 mm) were the second wettest on record with July 12 the wettest July day on record (51 mm). Heavy precipitation continued into August with 222 mm of precipitation falling (fifth wettest summer month on record) (Menne et al., 2012). These events created an extreme high‐frequency series of recurrent discharge peaks (Figure 1 Lemon Creek proximal to the study area). Significantly, these events followed an extreme winter flood in the same systems in November 2005 (Milner et al., 2013), with record rainfall (>650 mm in <72 hr) and widespread flooding across SE Alaska (>1 in 100 year flood). Contrasting the effects of these events provides a unique opportunity to understand how the timing and recurrence of extreme climate events will alter river ecosystems and their subsequent recovery.

The main aim of this study was to examine the effects of two contrasting extreme flood events one in the winter and one in the summer on riverine habitat and the associated biological communities in the context of a long‐term dataset. Specific objectives were to (a) examine whether the timing of the extreme events resulted in different biological effects, (b) assess how far each event reset the respective invertebrate communities, (c) determine the effect of the floods on the resilience of the different components of the biological community, and (d) develop a conceptual model of community response to extreme flooding events.

## 2 MATERIALS AND METHODS

### 2.1 Study area

In 1986, a continuous study was initiated of the ecosystem of Wolf Point Creek (WPC), a newly formed river sourced from a basin with ~70% glacial ice cover (58°59′49.84″N, 136°9′57.05″W) in Muir Inlet, Glacier Bay, Alaska. The mouth of WPC was uncovered by ice retreat in the mid‐1940s and the stream, fed from Lake Lawrence, is now approximately 2 km in length and flows over glacial moraine, till, and outwash deposits. Dolly Varden (*Salvelinus malma*) colonized the stream in 1987, followed by pink (*Oncorhynchus gorbuscha*) and coho (*O. kisutch)* salmon in 1989. Significant increases in stream temperature and decreases in turbidity were associated with continued decrease in glacial ice cover. By 1997 (<10% glacierization), alder (*Alnus* spp.) and willow (*Salix* spp.) were dominant with riparian plants exceeding 3 m in height and pink salmon numbering >12,000 individuals. In 2004, the glacial ice had almost completely disappeared and the upper terraces supported increasing numbers of cottonwood trees (*Populus trichocarpa*) along with the occasional Sitka spruce (*Picea sitchensis*). The watershed is now dominated by cottonwood with increasing abundance of Sitka spruce.

### 2.2 Channel profiles

Repeat channel cross section surveys were conducted in 2006, 2010, and 2016 using GPS referenced locations initially established in 1997. Once floodplain bank GPS locations were re‐located, a tape measure was extended from one bank to the other and fixed in place. Topographic height change from each floodplain bank was determined using a Sokkia dumpy level (Topcon, Tokyo, Japan), tripod, and staff. Floodplain height on the left bank was used as a control marker to account for differences in dumpy level setup, which allowed the cross sections to be comparable between years.

### 2.3 Salmon and invertebrates

Adult pink salmon spawners were estimated using the average of counts by two observers walking the length of the stream, and juvenile coho salmon densities were estimated with minnow traps baited with salmon eggs and fished for 2 hr. From 1986, macroinvertebrates (animals > 1 mm) were collected annually in August or early September randomly from a representative sampling station located 0.75 km from the stream mouth using a Surber sampler (10 replicates; 330‐μm mesh net) with the exception of 1987, 1995, and 2003. Following the 2005 extreme winter flood event, the site has been sampled every year until 2015 resulting in a cumulative total of 27 years of annual sampling events. From 1994, meiofauna (animals > 63 μm < 1 mm) were collected randomly during the same time period with the exception of 1995 and 1999 (where meiofauna were collected mid‐May) and 2005, 2009, 2011, 2012 (no sample). Samples were collected randomly from the same sampling station with a Surber sampler (five replicates; 63‐μm mesh net). All invertebrates were preserved in 70% ethanol and later separated in the laboratory from detritus and inorganic matter. Macroinvertebrates were identified using Merritt and Cummins (1988), and Chironomidae larvae were identified using methods outlined in Milner et al. (2000). Meiofauna were identified using Thorp and Covich (1994) and Smith (2001).

### 2.4 Statistical analyses

All statistical tests were completed using Minitab v15 or R v3.3.2 except Nonmetric Multidimensional Scaling (NMDS) which was undertaken using PRIMER v6 with each year included in the ordinations. Analyses were run with macroinvertebrate and meiofauna log_10_ (abundance + 1) data. Both analyses were conducted using Bray–Curtis dissimilarity matrices and 2000 restarts. Persistence was determined using the index of Jaccard (1912) and year pairs for both macroinvertebrates (23 pairs) and meiofauna (17 pairs). Nonparametric multivariate analysis of variance (PERMANOVA) tested the null hypothesis that differences in stream macroinvertebrate community composition between year groups before and after the flood (i.e., 1996–2005 vs. 2006–2008 vs. 2010–2013) were not different to those within year groups. Analyses were run using Bray–Curtis (BC) dissimilarity scores, with 10,000 permutations. Generalized least squares (GLS) regression of the two key chironomid species was applied to the time series of log_10_ transformed *Diamesa davisii* and *Pagastia partica* abundance after initial analysis revealed significant autocorrelation. Models took the form *P. partica* ~ *D. davisii* + e, where e = an error term modeled as a first‐order autoregressive process from the lag1 autocorrelation coefficient.

## 3 RESULTS

### 3.1 Channel profiles

**Figure 2:**
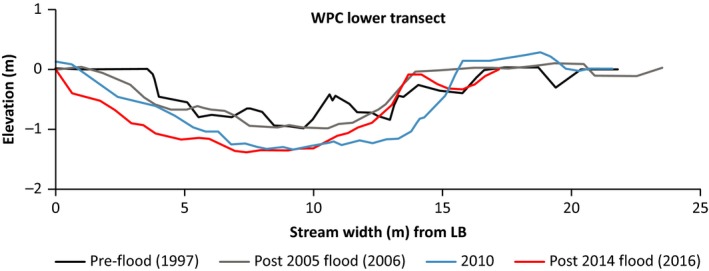
Cross sectional profiles of the WPC channel following both flooding events. (LB = Left Bank). Elevation is denoted in relation to a fixed bankside datum

A comparison of the WPC channel cross section at a long‐term sampling site before and after the 2005 flood indicated channel width had decreased from 22 to 12.1 m and had become incised by up to 1.1 m from active channel surface. Up to 0.6 m of sediment was deposited where water originally flowed (Figure 2). The channel has not widened postflood and has continued to deepen, particularly after the 2014 flooding (Figure 2). Pre‐2005 the wider channel supported a variety of flow types including slower flowing (pool and glide) areas (Klaar, Maddock, & Milner, 2009) but since 2006 faster flowing habitats (riffle, run) have dominated the WPC sampling site.

### 3.2 Salmon

Sediment erosion and deposition following the 2005 flood caused considerable mortality to incubating pink salmon eggs, such that estimates of returning pink salmon in 2007 (returning adult spawners from the 2005 egg deposition—2‐year life cycle) were <500 compared to the >14,000 estimated in late summer 2005 before the flood. However, in 2011 (i.e., within two generations), pink salmon spawners had recovered to preflood levels, exceeding 14,000 (Milner et al., 2013). The summer flooding in 2014 occurred principally before, and partially during, the peak spawning of pink salmon and in 2016 the number of spawners from the cohort of eggs laid in 2014 exceeded 8,000 (Table 1). The average number of spawners for 2010 and 2012 was 7,000. Juvenile coho salmon densities were reduced significantly from a mean catch per unit effort (CPUE) of 9.3 (2003–2005) to 0.6 in 2006 after the winter flood. Densities remained low in 2007 (<1 CPUE) but recovered to a mean CPUE of 6.6 juvenile coho salmon for the years 2008–2010. In 2015, CPUE was reduced to 0.4 from 2.8 in 2013 and 3.2 in 2014 and was similar in 2016 at 0.58 (Table 1).

**Table 1:** Adult pink salmon spawner estimates and juvenile coho salmon abundance in Wolf Point Creek 2004–2016. (Adult counts were not possible every year due to high flows)

Year	Adult pink salmon spawners	Juvenile coho salmon (CPUE)
2004	4500	10.2
2005	>15,000	8.3
2006	No count	0.6
2007	<500	0.8
2008	5121	10.6
2009	6120	4.8
2010	6500	4.3
2011	14,130	9.3
2013	No count	2.8
2014	7200	3.2
2015	No count	0.4
2016	>8,000	0.58

### 3.3 Invertebrate community structure

**Figure 3:**
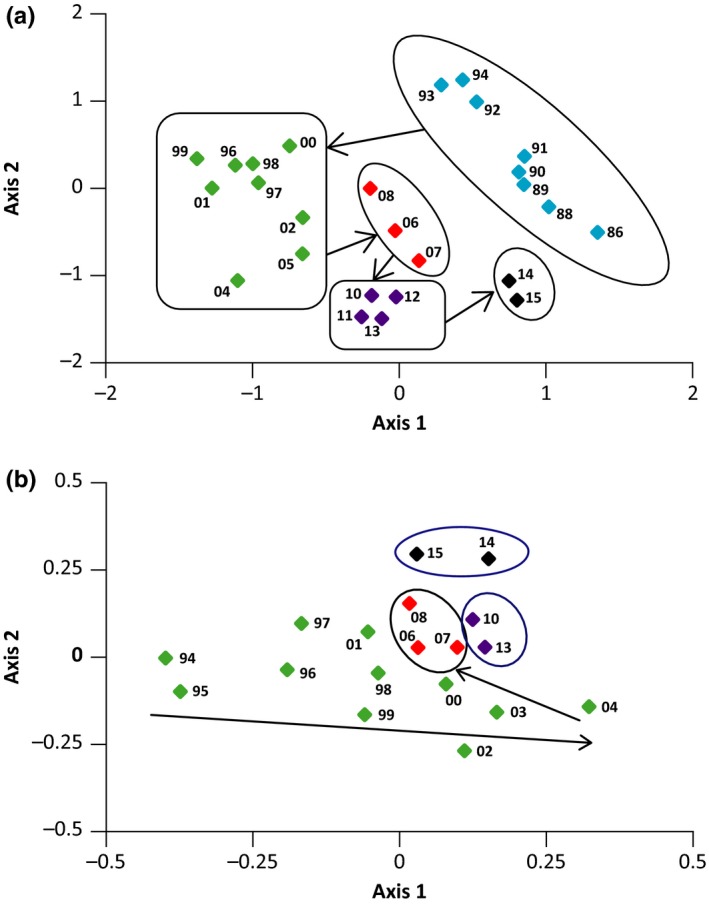
(a) NMDS plots for macroinvertebrates from 1986 to 2015 using mean abundance data from ten replicates collected in August/early September and (b) meiofauna from 1994 to 2015 using mean abundance data from five replicates collected during the same time period. The immediate postflood years are outlined in red and black and the arrows highlight significant shifts in the macroinvertebrate community structure between the groups of years. Numbers represent years

Nonmetric Multidimensional Scaling (NMDS) analysis of changes in abundance and addition/loss of taxa within the macroinvertebrate community over the 30 years indicated two preflood successional groups (Figure 3a): (a) the years 1986 to 1994, and; (b) the years 1996 to 2005 due to the extinction of early colonizing taxa with increased water temperature and potential competition (Brown & Milner, 2012; Flory & Milner, 1995). Additionally in the period 1996–2005, the chironomid *Chaetocladius*, the caddisfly *Ecclisomyia*, and the families Gammaridae, Dysticidae, and Ceratopogonidae colonized and Simuliidae became more abundant. Immediately after the major winter flood of 2005, the years 2006–2009 showed a distinct “reset” of the macroinvertebrate successional community with the years on axis 1 lying between these two groupings. A number of chironomid taxa (see Discussion below) and the predatory stonefly *Suwallia tibialis* were not collected after 2005 and Dysticidae, Gammaridae, Planorbidae, and Corixidae, taxa typical of slower flowing habitats, were also eliminated (Table 2). During subsequent years (2010–13), the community did not recover toward a pre‐2005 composition but to another grouping of the community toward the negative region of axis 2 (Figure 3a labeled in purple). PERMANOVA analysis indicated the community structure of these groups was significantly different (*p *<* *0.05). After the recurrent summer flooding of 2014, the community was reset markedly again; this reset was similar to the one following the 2005 flood in that the community composition shifted toward the positive region of axis 1, although it was more similar to the early successional community of 1986–1994 than previously (Figure 3a). The chironomids *Tanytarsus, Eukieferiella devonica,* and *Chaetocladius* were not found (Table 2).

**Table 2:** Taxa eliminated and colonizing following the two flooding events and the number of years newly colonizing taxa persisted in the community following these events is given in brackets

Flood event			
2005		2014	
Taxa not collected after flood	Taxa colonizing (no of years present after flood)	Taxa not collected after flood	Taxa colonizing (no of years present after flood)
*Suwallia tibialis*			
	*Diamesa davisi (1)* *Neuropertona*	*Tanytarsus* *Eukieferiella devonica* *Chaetocladius*	*Diamesa davisi (1)*
Dysticidae	*Maraenobiotus brucei (3)*		Ostracoda (2)
Planorbidae	*Bryocamptus zschokkei*		*Maraenobiotus brucei (1)*
Gammaridae	*Chydorus (2)*		
Corixidae	*Pleuroxus (3)*		
Ostracoda	*Chaetogaster*		
*Cyclops scutifer*			

From 1994 to 2004, the successional trajectory of the meiofaunal community shifted to the positive region of axis 1 of the NMDS (Figure 3b). NMDS analysis suggested that the meiofaunal community showed similar responses to both the winter 2005 and the summer 2014 flooding episodes. Following both events, the community reset back along axis 1 (although this was less marked following the 2014 floods) and formed a new grouping in the positive region of axis 2 (Figure 3b). This reset was primarily driven by increases in the abundance of cyclopoid copepods and the annelid *Chaetogaster* and the recolonization of the harpacticoid copepods *Maraenobiotus brucei* and *Bryocamptus zschokkei* (Table 2).

**Figure 4:**
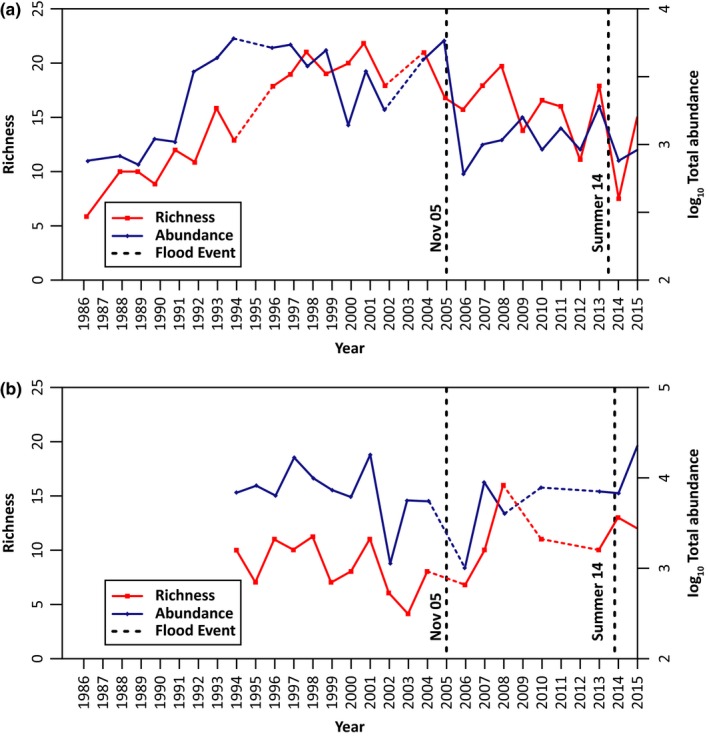
Taxonomic richness and total abundance of macroinvertebrates and meiofauna over time. (a) macroinvertebrates from 1986 to 2015 using mean numbers from ten replicates collected in August/early September; and (b) meiofauna from 1994 to 2015 using mean numbers from five replicates typically collected in summer. Vertical dashed lines indicate the approximate timing of the two flood events and represent a flood occurring after that particular year's sampling

Macroinvertebrate taxonomic richness increased from six in 1986 to 13 in 1994 during the first 1986–1994 successional trajectory and then to 21 in 1998 where richness oscillated between 17 and 23 until the winter flood in 2005. Taxonomic richness was not markedly reduced by the winter flood but never recovered to preflood richness peaks. Following the 2014, summer flooding richness decreased from 16 to 11, similar to a level last observed in 1992. Total macroinvertebrate abundance was reduced to a greater extent by the major winter flood (by an order of magnitude) than the atypical summer flooding (Figure 4a). In contrast, the meiofaunal community increased in abundance following each flood period. Higher abundance of permanent meiofauna (taxa that remain within the meiofaunal size range throughout their life cycle) was found as compared to early instar macroinvertebrates, such as Chironomidae (Figure 4b). For example, the mean density of the cyclopoid copepod *Acanthocyclops vernalis* increased from a mean of 6 in 2004 to 1,480 m^−2^ postflooding in 2006 and from 9 in 2013 to 4,566 m^−2^ postflooding in 2014. This species inhabits both river and lake benthos (Paterson, 1993; Robertson, Lancaster, & Hildrew, 1995) and the greater densities postflooding may reflect increased connectivity between the upstream lake and the stream channel.

**Figure 5:**
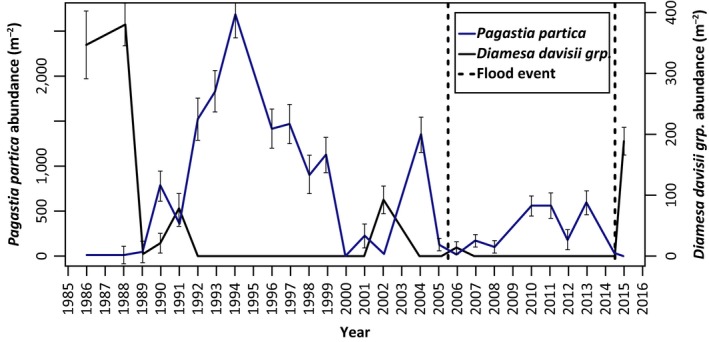
Comparison of the abundance of *Diamesa davisii* grp. and *Pagastia partica* (+/− 1*SD*). A significant negative relationship (*p* < 0.01) was evident from a generalized least squares (GLS) regression of log_10_ abundance

The immediate postflood changes in the macroinvertebrate community from both flood events showed distinct shifts in the Chironomidae assemblage with a number of taxa going extinct after the 2014 flood. *Diamesa davisii* grp., which favors early successional river habitats, declined in abundance from 1990 as other chironomids colonized the river and became extinct in the late August community of WPC in 1992. However, this species notably recolonized in 2002 (wettest summer on record with 307 mm rain in August including 50 mm in 1 day resulting in high flows >50 m^3^s (Menne et al., 2012), 2006 (following the winter flood) and in 2015 (following the summer flooding). The abundance of *D*. *davisi* was significantly related to the low abundance of a potential competitor *P. partica* (Flory & Milner, 1995) postflooding (Figure 5). However, by 2008, as the abundance of *P. partica* and other chironomid taxa recovered, *D. davisi* were again not collected in the WPC community.

### 3.4 Invertebrate persistence

The largest decrease in macroinvertebrate persistence followed the recurrent 2014 flooding with only 24% similarity compared to 54% similarity following the 2005 flood reset (see Appendix Table 1). After the 2014 floods, a number of chironomid taxa were eliminated which had persisted through the earlier extreme flood and this group would appear to have been more susceptible to the atypical summer flooding than the 2005 winter flood. The chironomid *Paratrichocladius* was the only taxon that has been found over the entire 30‐year record and has persisted through all the major flood events.

Persistence of the meiofaunal community decreased to a similar extent following both floods (46% similarity compared to 75% and 66% in preceding year pairs) (Appendix Table 1). However, the meiofaunal community was resilient following both floods in that total abundance of the meiofaunal community showed negligible change. Taxon richness increased as previously unrecorded species became established in the community and other species recolonized after an absence of two or more years. Nevertheless, the composition of the community changed following flooding; abundance of permanent meiofauna increased, whereas that of early instar macroinvertebrates, such as Chironomidae, decreased. Overall the turnover of the meiofaunal community was smaller following the summer 2014 floods (two taxa recolonizing after noncollection for at least 2 years) than after the 2005 winter flood (five taxa recolonizing and two were not collected), suggesting the impact of recurrent summer flooding on the meiofaunal community resilience was less than for the macroinvertebrates, perhaps because reproduction rates were high in response to relatively high water temperature (Dole‐Olivier et al. 2018).

## 4 DISCUSSION

The magnitude of extreme flooding events is predicted to increase in the future with climate change, while the predictability of peak flow timings is likely to decrease (Ledger & Milner, 2015). Our study demonstrates that the magnitude and direction of ecological impacts can vary according to the timing and extent of the extreme flood event. The November 2005 flood occurred when Pacific pink salmon eggs were incubating in the gravel resulting in the low 2007 adult salmon return. However, the summer flooding in 2014 occurred before the peak of pink salmon spawning and the majority of eggs had not yet been deposited in the gravel redds. Hence, numbers of pink salmon spawners 2 years later were unaffected. Indeed, salmonid spawning may be improved if flooding takes place when eggs are not in the gravels because flooding can remove finer sediments (George et al., 2015). Total macroinvertebrate abundance was reduced by an order of magnitude more by the winter flooding than the recurrent summer flooding. A major influential factor accounting for this finding was life history stage; in winter, juvenile stages of all insect species inhabit the stream bed. Recolonization following egg deposition by adult stages is low to nonexistent as few adults emerge at this time of year due to the stream being potentially ice covered in this part of Alaska. Thus, the potential for rapid recolonization (and thus overall community resilience) may be reduced when extreme events occur in winter.

**Figure 6:**
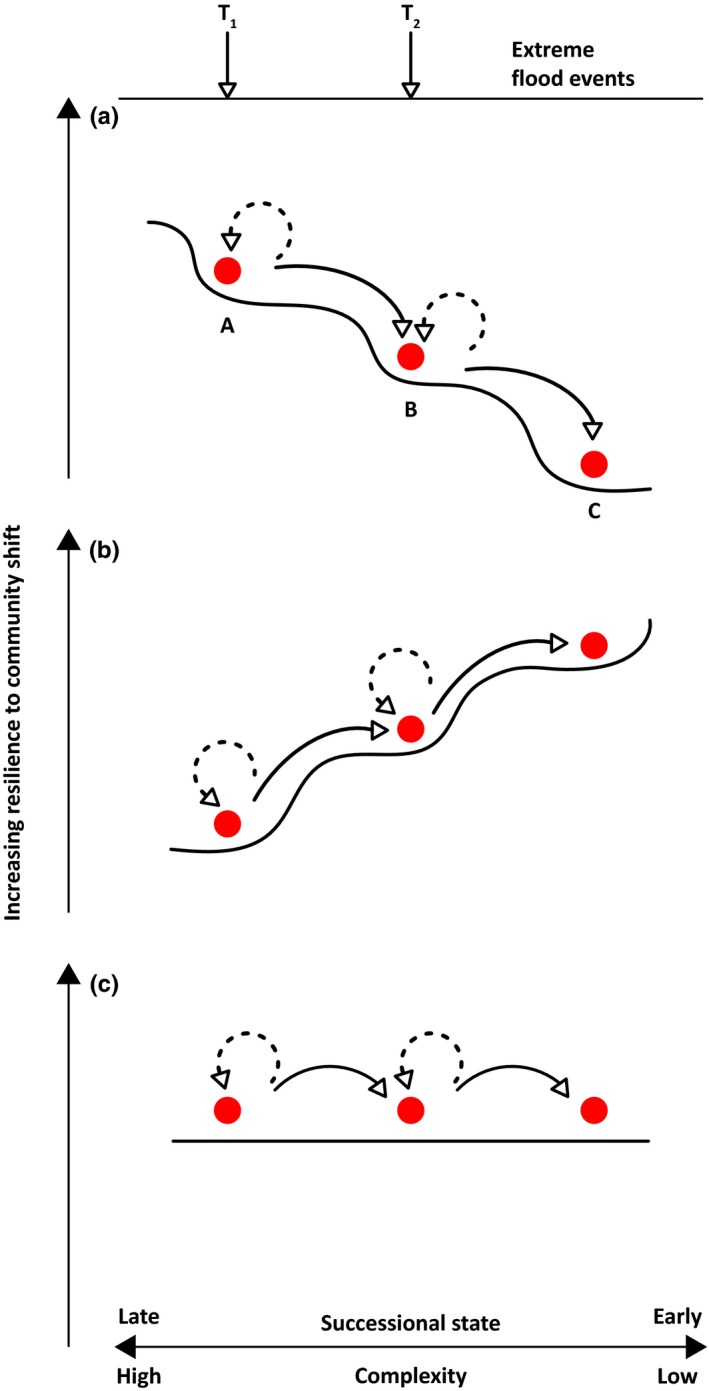
Conceptual changes in river invertebrate communities following two extreme flooding events occurring at different times T_1_ and T_2_. Event T_1_ may shift a community from state A to state B which is less complex and at an earlier successional stage, and therefore (a) less resilient, (b) more resilient, or (c) unchanged in resilience to a further extreme event at T_2_ (see text)

Persistent shifts in overall macroinvertebrate community structure were evident after the 2005 flood even after 3 years of recovery. Subsequent annual investigations to 2013 showed that the community had not recovered to the pre‐2005 flood state. The 2014 flooding appears to have further altered the community structure of Wolf Point Creek into an alternative state. These findings suggest different end‐points from the two contrasting extreme flooding regimes, perhaps due to the community not recovering fully from the major flood event in 2005 before the 2014 atypical summer flooding. The meiofaunal communities also showed a marked reset with lower complexity and an earlier successional state following the first flood. However, the responses of the two communities to the second flood then diverged. Macroinvertebrate community persistence was lower during the second flood compared to the first, whereas the meiofaunal community persistence during the second flood was very similar to that following the first flood. Extreme climate events can drive catastrophic shifts in ecosystems (Scheffer, Carpenter, Foley, Folke, & Walker, 2001), and findings from this study suggest alternative state theory can be adopted to propose a conceptual framework (Figure 6) of how two differing extreme flooding events can influence a riverine invertebrate community during successional change and may cause a shift to an alternative state where the community is more or less resilient to future extreme flooding events. Our proposed framework should then be tested more widely with data from multiple areas and flood events.

Over time following glacial recession communities show a central successional tendency to shift toward state A. Consider then two extreme flooding events, potentially occurring at different times of the year, either in the same year or in different years. The first extreme flooding event (T_1_)_,_ can have two outcomes (1) a shift from state A to state B (evidenced by solid lines) or (2) the community is resilient to change either through initial resistance or rapid recovery (as evidenced by dotted lines) and remains at state A. A shift to state B in outcome 1 can then have three potential effects on the resilience of the community to a further extreme flooding event at T_2_ —in scenario (a), the shift makes the community less resilient and will more easily reset further to state C which is less complex and similar to an early successional stage (i.e., increased likelihood of following solid line trajectory; Figure 6a), or in scenario (b) the shift makes the community more resilient to further change and thus more difficult for an extreme event to shift the community from B to C (i.e., increased likelihood of following dotted line trajectory; Figure 6b), or in scenario (c) the shift to state B does not affect the resilience of the community to future flooding events and is similar in resilience to state A (Figure 6c).

The traits possessed by taxa comprising the community will inevitably influence community response to extreme flooding. Certain traits may confer initial resilience to extreme flooding (i.e., the community remains at state A; outcome 2) or following flooding and outcome 1 such traits may be more common in the alternative state community so that after further flooding, the community is more resilient to disturbance and less likely to shift to state C (i.e., outcome 1b). For example, the trait of small body size may confer or improve resilience in hydraulically turbulent conditions (Segura, Siqueira, & Fonseca‐Gessner, 2014; Snook & Milner, 2002) because smaller body size facilitates access to refugia during high flows following storm events (Mesa, 2012). The persistence of a number of the smaller chironomid species following the extreme events documented here supports this idea, and small body size could also account for the observed resilience in the meiofaunal community following the two flooding events (Robinson, 2012). In addition, some species have developed traits related to life history evolution to avoid predictable large magnitude flooding which include fast development to aerial adult stage and egg‐laying synchronized with seasonal lower flows (Lytle & Poff, 2004; Southwood, 1977).

Where the community becomes more resilient to future change following a shift to a different state (Figure 6b), a further shift from state B to state C may require an extreme event (T_2_) that is higher intensity (size or duration) and/or has differing timing and predictability than the first event. The adaptations outlined earlier that confer resilience may only be invoked when the event happens “predictably” at specific times of the year relative to an organism's life cycle. Timing of the flooding event may thus be critical; for example, during the summer, many aquatic insects are in their aerial stage and can lay their eggs rapidly following a flood, whereas in winter, the potential for aerial colonization is limited as the majority of insects are in the egg or larval stage and are therefore potentially lost during the flood. The order of magnitude difference in reduction of total macroinvertebrate abundance following the November flooding event compared to the summer flooding events could thus relate to life history. The effect of a summer extreme flooding event may also be reduced for taxa that undergo continuous reproduction, as is the case for many meiofauna, because higher temperature results in rapid population increases and therefore greater resilience.

In addition, long‐term changes in the habitat template (Gothe et al., 2017) as well as dispersal limitations (Brown et al., 2018) can restrict recovery of ecosystems following disturbance. In the case of WPC, the physical habitat template has not regained the slower flowing habitats evident before the 2005 flood and thus recolonization by Dysticidae, Gammaridae, Planorbidae, and Corixidae had not occurred by 2015. While there may be dispersal limitation effects impeding the recolonization of these groups, populations are present in nearby ponds and therefore the lack of geomorphological recovery would appear to be a major reason for their continued absence in the river. Conversely, increased sedimentation after the 2014 flood in some areas of the river has enhanced the abundance of Oligochaetae worms and may have impacted some of the chironomid species following the summer flooding causing overall lower community persistence. The lack of resilience of some groups and the shift in community states is undoubtedly linked to the (potentially long term) changes in the habitat template caused by the flood events. The findings clearly demonstrate fugitive taxa, like *D. davisii* grp, depend upon major disturbances to maintain populations in rivers illustrating the role of extreme flood events in enhancing river biodiversity. This taxon can also provide an indication of potential past extreme flood events when they appear in the record. Other groups were surprisingly resilient; juvenile coho salmon recovered rapidly following the 2005 flood despite the continued lack of geomorphological complexity in the stream with respect to their preferred pool habitat. This finding bolsters our previous suggestion that markedly different responses according to the organismal group mean that caution is required when applying ecosystem theories and concepts to predict responses to flood events at the whole river ecosystem scale (Milner et al., 2013).

Our findings have significant implications for the management of rivers to conserve biodiversity in light of increased incidences of extreme flood events. The implementation of mitigation strategies such as the preservation of remnant population refuges and proximal colonizing courses may be more critical according to the timing of the event, especially if floods occur during the winter or early spring rather than the summer. Continued disturbances may necessitate restoration of geomorphic complexity (i.e., the physical habitat template), because the natural recovery of complexity is very slow. This approach could facilitate the recolonization of taxa that have been eliminated and enable the reversal of the documented shifts in community states following these extreme events. More realistically, managers must accept dynamic change as a natural component of river ecosystems (Mainstone, 2017) and rivers should be allowed to flood and rework their morphology and biodiversity, and allow the persistence of fugitive species. However, where extreme events occur during system recovery (e.g., river restoration) from previous disturbances that have caused reduced biodiversity, then restoration practices that increase the resilience of the system to these events may need to be implemented to prevent prolonged extensions to recolonization periods (Reich & Lake, 2015).

## CONFLICT OF INTEREST

None declared.

## AUTHOR'S CONTRIBUTIONS

A.M.M. initiated the study of WPC and collected many of the preflood samples. A.M.M., A.L.R., M.J.K., and L.E.B were responsible for the funding applications to study the postfloods ecosystem, research design and planning, data collection and analysis, and writing the manuscript. J.P. and LC assisted with fieldwork and analyzed the postflood samples in the laboratory. J.P. and L.E. undertook statistical analyses and assisted with writing the manuscript.

## DATA

Data created during this analysis are available from the University of Birmingham ePapers repository at http://epapers.bham.ac.uk/.

## APPENDIX 1

### Table A Percent Jaccards Similarity Coefficients for year pairs for the macroinvertebrate and meiofaunal communities

**Table:**

Year pair	Macroinvertebrates	Year pair	Meiofauna
1986–1988	60		
1988–1989	82		
1989–1990	90		
1990–1991	50		
1991–1992	77		
1992–1993	59		
1993–1994	71		
1994–1996	48	1994–1995	70
1996–1997	76	1995–1996	28
1997–1998	67	1996–1997	46
1998–1999	74	1997–1998	58
1999–2000	77	1998–1999	64
2000–2001	91	1999–2000	66
2001–2002	67	2000–2001	80
2002–2004	46	2001–2002	54
2004–2005	60	2002–2003	71
2005–2006	54	2003–2004	75
2006–2007	59	2004–2006	46
2007–2008	39	2006–2007	75
2008–2010	40	2007–2008	62
2010–2011	83	2008–2010	58
2011–2012	82	2010–2013	66
2012–2013	77	2013–2014	46
2013–2014	79	2014–2015	66
2014–2015	24		
